# A corpus and a concordancer of academic journal articles

**DOI:** 10.1016/j.dib.2017.11.023

**Published:** 2017-11-08

**Authors:** Deny A. Kwary

**Affiliations:** English Department, Faculty of Humanities, Universitas Airlangga, Jl. Dharmawangsa Dalam, Surabaya 60286, Indonesia

## Abstract

This data article presents a corpus (i.e. a selection of a big number of words in an electronic form) and a concordancer (i.e. a tool to show the word in its context of use) of academic journal articles. As the title suggests, the data were collected from research articles published in academic journals. The corpus contains 5,686,428 words selected from 895 journal articles published by Elsevier in 2011–2015. The corpus is classified into four subject areas: Health sciences, Life sciences, Physical Sciences, and Social Sciences, following the classifications of Scopus, which is the largest abstract and citation database of peer-reviewed scientific journals, books and conference proceedings. To ease the access and utilization of the corpus, a program to produce the key word in context (KWIC) and word frequency was created and placed on the website: corpus.kwary.net. The corpus is a valuable resource for researchers, teachers, and translators working on academic English.

**Specifications Table**

**Table:** 

**Subject area**	*Linguistics, Education, English Language*
**More specific subject area**	*Corpus Linguistics, Adult Education, English Language Teaching, English Language Learning, Academic Writing*
**Type of data**	*Texts and tables*
**How data was acquired**	*Select the articles that meet the pre-determined criteria, download the articles, convert the texts into a.txt format, clean the corpus, and create the concordancer using PHP and MySQL.*
**Data format**	*Raw and processed*
**Experimental factors**	*The journals were classified into four subject areas: Health sciences, Life sciences, Physical Sciences, and Social Sciences, following the classifications of Scopus. The pre-determined criteria for the selection are: (1) The journals do not appear in more than one subject area, (2) the journals have a 5-year impact factor, (3) the articles are published in 2011 and 2015, (4) the articles are written in English, and (5) the articles are open access.*
**Experimental features**	*The corpus comprises well-selected and recent journal articles. The concordancer enables the search of particular words to determine its context of use.*
**Data source location**	*The data were gathered from the website:*sciencedirect.com
**Data accessibility**	*Free online access at corpus.kwary.net*

**Value of the data**

• Corpus linguists and English lecturers will be able to compare the results derived from these corpus and concordancer with the data found in other studies or textbooks.
• Researchers from the four subject areas (Health sciences, Life sciences, Physical Sciences, and Social Sciences) will be able to know the particular words which are frequently used in the recent journal articles in their own subject areas.
• Researchers from the four subject areas will be able to know how extensive a particular term or topic has been written in the recent journal articles in their own subject areas.
• Teachers of academic English will be able to know the common collocates of a particular word when it is used in journal articles.
• Translators will be able to select the right words and collocates when translating a particular word from a source language to English.

## 1 Data

The corpus comprises 5,686,428 words, classified into four subject areas: Health sciences, Life sciences, Physical Sciences, and Social Sciences, following the classifications of Scopus. The words were compiled from 895 journal articles published by Elsevier in 2011–2015. To ease the access and utilization of the corpus, a concordancer to produce the key word in context (KWIC) and a word frequency tabulation were created and placed on the website: corpus.kwary.net. When we open that website, we will be able to search for a word or several words to see the KWIC which is the position of the word together with its collocates (up to five word tokens to the left and five tokens to the right). There is also a link on the left-hand top of the page to access the frequency list, i.e. corpus.kwary.net/freq. This is useful to make a word list of the words found in each discipline.

## 2 Experimental design, materials and methods

A corpus as a collection of pieces of language text in electronic form, selected to represent a language or language variety [1]. A corpus enables the elaboration of better quality learner input and provides researchers and teachers with a wider, finer perspective into language in use [2]. The corpus available here is expected to be a resource for determining the behaviour of the words used in research articles published in international journals. Consequently, the data were selected from international journal articles. However, it is necessary to note that the behaviour or the usage of a particular word in one discipline can be different from that in another discipline. An analysis of the text from the British National Corpus shows that there are differences between the usage of the word *admit* in medical and in commerce [3]. Consequently, the corpus presented here also needs to be classified into several groups.

There are several possibilities to classify the subject areas or disciplines. The one selected here is the classification made by Scopus. This is because Scopus is the largest database of academic literature. Following Scopus's classifications, the subject areas in this corpus are classified into Health sciences, Life sciences, Physical Sciences, and Social Sciences. As a large database, Scopus comprises a number of publishers, and the publisher that has the biggest number of data is Elsevier. Therefore, the data in this corpus were taken from research articles published by Elsevier.

Elsevier also categorizes the subject areas into four. Two of them are exactly the same as those used in Scopus, i.e. health sciences and life sciences. The other two are quite similar to those of Scopus, i.e. Elsevier uses the terms ‘social sciences and humanities’ and ‘physical sciences and engineering’, whereas Scopus uses the terms ‘social sciences’ and ‘physical sciences’, respectively. In this corpus, as mentioned earlier, the titles of the subject areas used are as follows: Health sciences, Life sciences, Physical Sciences, and Social Sciences.

In selecting the research articles to build the corpus, five criteria were taken into account. The first criterion is that the journals do not appear in more than one subject area. As we know, some journals can be interdisciplinary in nature. Such journals are not included in this corpus, because the corpus is aimed to present the data which reflect the uniqueness of each subject area. Consequently, only journals that appear in only one subject area were selected. In addition, to ensure equal distributions of the corpus across different subject areas, only one article is selected from each journal.

The second criterion is that the journals must have a 5-year impact factor. Having at least a 5-year impact factor means that journals have been established for more than five years and that their articles have also been cited in other research articles for more than five years. This means that the journals have established a sufficient authoritative position in the subject areas, and have shown prominent academic quality.

The third criterion is that the articles selected from those journals are the articles published in 2011 and 2015. Language changes over time, so the language items used in the past can be different from those used nowadays. Therefore, it is necessary to take the data for the corpus from the recent use of the language. In this case the data from 2011 to 2015 are considered the representation of the current use of language, since the corpus was started to be created in mid-2016.

The fourth criterion of the selection is that the articles must be written in English. International journal articles can be written in several language, e.g. English, French, and Spanish. However, most journal articles are written in the English language, which has been established as an international language for a number of years. Consequently, the corpus created here only took into account the articles written in English. These articles could have been written by non-native speakers of English, but they are still included in the corpus because the focus of the data is on the English language used in international journal articles. In addition, articles which have been published in an international journal with a good impact factor (cf. the second criterion above), should have used the standard English language.

The fifth criterion is that the articles must be open access articles. Being open access means that the research articles can be accessed and downloaded for free. This is necessary to make the collected data to be copyright free to be distributed via this corpus. Based on these five criteria, we found 895 articles to build the corpus. The details can be seen in Table 1.

**Table 1:** The details of the corpus.

**Subject Areas**	**Number of Running Words**	**Number of Research Articles Selected**	**Sub-disciplines covered (based on Elsevier's classifications)**
*Health Sciences*	1,094,205	246	(1) Medicine and Dentistry, (2) Nursing and Health Professions, (3) Pharmacology, Toxicology and Pharmaceutical Science, (4) Veterinary Science and Veterinary Medicine.
*Life Sciences*	987,279	161	(1) Agricultural and Biological Sciences, (2) Biochemistry, Genetics and Molecular Biology, (3) Environmental Science, (4) Immunology and Microbiology, (5) Neuroscience.
*Physical Sciences*	2,632,817	366	(1) Chemical Engineering, (2) Chemistry, (3) Computer Science, (4) Earth and Planetary Sciences, (5) Energy, (6) Engineering, (7) Materials Science, (8) Mathematics, (9) Physics and Astronomy.
*Social Sciences*	1,040,259	122	(1) Arts and Humanities, (2) Business, Management and Accounting, (3) Decision Sciences, (4) Economics, Econometrics and Finance, (5) Psychology, (6) Social Sciences.
**Total**	**5,754,560**	**895**	

After downloading the articles that meet the selection criteria, the downloaded files (which are in pdf format) were converted into txt file. This is necessary since a corpus program can only access data in the form of txt. The txt files were then uploaded to the website corpus.kwary.net. Then, to ease the access to the corpus data, a program to produce the KWIC and word frequency was created by using PHP (Personal Home Page/Hypertext Preprocessor) and MySQL (My Structured Query Language).

The KWIC shows the use of a word in the context of five words to its left and five words to its right. The adjacent collocate, i.e. the collocate that occurs immediately to the right or to the left of the keyword, is tabulated, because an adjacent collocate is closer to real linguistic structures [4]. In this corpus, the tabulation of a keyword's adjacent collocates is presented in two tables: one comprises the adjacent collocates to the left of the keyword, and the other contains the adjacent collocates to the right of the keyword. These will make it easier for teachers and researchers to see the behaviour of the keyword, i.e. knowing what words usually come before and after the keyword.

**Fig. 1:**
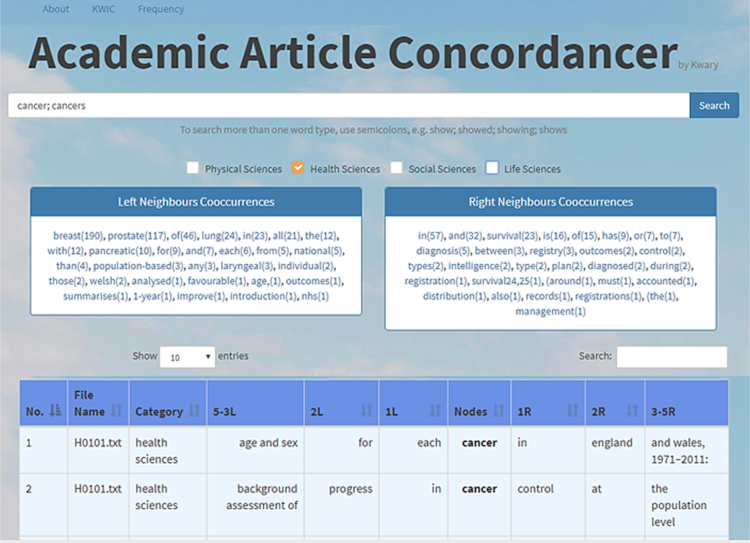
Search Result for the word *cancer* and *cancers* in Health Sciences.

The keyword may consist of one word or more. This means that we can search for several words directly, by using a semicolon and a space to separate one word with another. For example, if we want to know how the words *cancer* and *cancers* have been used in the Health Sciences journal articles, we can type both words (*cancer; cancers*) in the search box on the top of the screen, un-tick the boxes of the other three disciplines (i.e. leaving only Health Sciences’ box ticked), and press Enter or click Search. The result is shown in Fig. 1.

As shown in Fig. 1, the concordancer enables us to see the behaviour of the keywords (in this case, they are *cancer* and *cancers*). Fig. 1 only presents the first two entries of the KWIC, but if we scroll it down, we will be able to find a total of 803 entries. The tabulation of the adjacent collocates are presented in two tables. The first table—Left Neigbours Cooccurrences—contains the list of words which occur on the left of the keywords, together with the frequencies of the words. In this case, we can see, for example, that the phrase *breast cancer(s)* occur more often than the phrase *prostate cancer(s)*, which is 190 against 117. Then, the third type of cancer(s) which is often discusses in the research articles is *lung cancer(s)* with a frequency of 24.

**Fig. 2:**
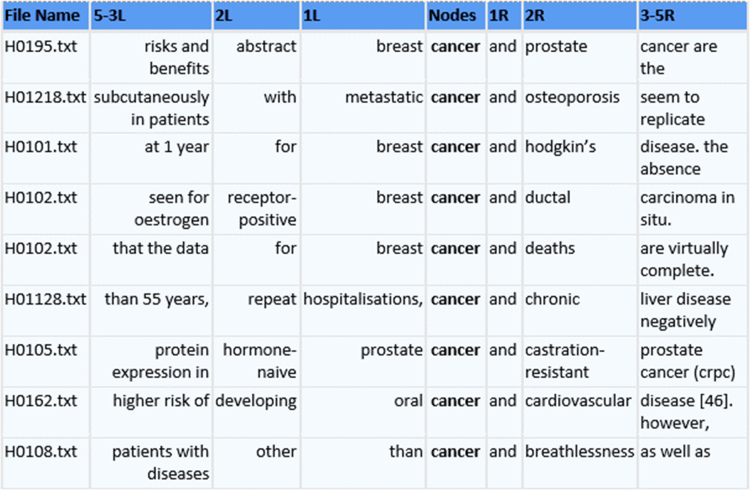
Search Result for the word *cancer(s)* and the word *and* in Health Sciences.

The second table—Right Neigbours Cooccurrences—comprises the list of words which occur on the right of the keywords, together with the frequencies as well. In this case, we can see that the most common preposition used after *cancer(s)* is *in*, which is 57 times. We can also see that *cancer(s)* is sometimes used with the conjunction *and* (i.e. 23 times). If we click on the word *and*, we will be able to see the examples where the *cancer(s)* is used with *and*. Fig. 2 presents some of the results when the word *cancer* or *cancers* is used with the word *and*. In that figure, we can see what types of cancers are mentioned together, i.e. breast cancer and prostate cancer, and what kinds of diseases are mentioned when *cancer(s)* is mentioned, i.e. metastatic cancer and osteoporosis, breast cancer and Hodgkin's disease, etc.

Another example of a difference in the word usage, for Physical Sciences and Social Sciences, is the word modified by the adjective *social*. In the Physical Sciences, the nouns most frequently modified by the word *social* are *media* and *networks*, whereas in Social Sciences, the nouns are *networks* and *interactions*. These show that both articles in Physical Sciences and those in Social Sciences discuss *social networks*. However, articles in Physical Sciences focus more on the media, while those in Social Sciences focus more on the interactions.

In addition to the KWIC, the program in this website can also produce word frequency lists. There are several functions of a word frequency list, one of them is to show learners which words are most worth studying [5]. An example of a study which has used the frequency list data is the article which formulates the Academic Article Word List for Social Sciences [6].

**Fig. 3:**
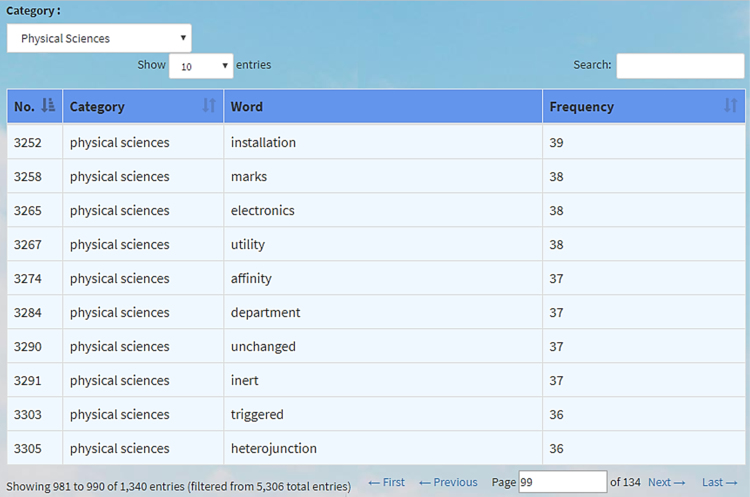
Word Frequency List of the Words in Physical Sciences.

The frequency list menu can be accessed by clicking Frequency on the left hand top of the screen, or by opening the following web page: corpus.kwary.net/freq. In this web page, we can make a list of all words found in the corpus, together with their frequencies. We can also make a list of the words found in each category. For example, if we want to see the list of the words used in Physical Sciences, we can choose Physical Science under the Category (see Fig. 3). At the bottom of the web page, we can choose which words to be shown. In Fig. 3, the number 99 was typed in the text box near the Page, so the results show the words which are on page 99 of the list of 134 pages. The frequency of each word is also presented. In this case, we will be able to see the typical words used in that category. This means that a further analysis comparing the words used in one category or discipline with those used in another discipline can be made.

## Funding sources

This work was supported by Research Mandate Program from the Rector of Universitas Airlangga [Grant number: 549/UN3.14/LT/2016].
